# Automated cytotoxicity assessment of natural killer cells by flow cytometry

**DOI:** 10.3389/fimmu.2026.1868673

**Published:** 2026-06-29

**Authors:** Aleksander Szarzynski, Valentin von Werz, Gregor Mattert, Werner Dammermann, Oliver Spadiut

**Affiliations:** 1 Research Area Biochemical Engineering, Institute of Chemical, Environmental and Bioscience Engineering, TU Wien, Vienna, Austria; 2 Center for Translational Medicine Germany, University Hospital Brandenburg, Brandenburg Medical School Theodor Fontane, Brandenburg an der Havel, Germany; 3 Faculty of Health Sciences Brandenburg, Brandenburg an der Havel, Germany; 4 Department of Gastroenterology, Diabetology and Hepatology, Center for Internal Medicine II, University Hospital Brandenburg, Brandenburg Medical School Theodor Fontane, Brandenburg an der Havel, Germany

**Keywords:** automatization, cytotoxicity, flow cytometry, gating strategy, NK cells, NK-92 cell line

## Abstract

Natural killer (NK) cell-based therapies are emerging as highly promising candidates for cancer treatment, but their development and quality control depend on robust assessment of key critical quality attributes, particularly their cytotoxicity. Despite the availability of various approaches for assessing cytotoxicity, existing techniques often suffer from high data variability and show limited reproducibility. We compared commonly used approaches for NK-cell cytotoxicity assessment, including calcein release, lactate dehydrogenase release, and flow cytometry (FCM)-based analysis, using NK-92 effector and GFP-labelled K562 target cells. Our results provide insight into the shortcomings of these methods, as well as problems resulting from unharmonized evaluation criteria and limitations of endpoint measurements, which are commonly applied in literature. We selected FCM as the most suitable platform for standardized evaluation and developed an automated gating workflow for cytotoxicity analysis. The automated workflow was benchmarked against three independent manual evaluations to assess agreement, bias, and performance. The optimized workflow showed agreement with manual analysis while remaining essentially unbiased. In addition, automated analysis reduced evaluator dependence by producing deterministic outputs from identical input data. Further comparison revealed directional bias in manual gating, indicating that a relevant portion of measurement variability arose from manual evaluation, rather than biology alone. We present autogating as a fit-for-purpose, automated FCM-based strategy for NK-cell cytotoxicity evaluation that preserves agreement with manual analysis while improving standardization and reproducibility, thereby providing a practical route toward more harmonized cytotoxicity testing in cell therapy workflows.

## 1 Introduction

Natural killer (NK) cells are cytotoxic lymphocytes of the innate immune system that play a central role in the recognition and elimination of malignant or virus-infected cells. Physiologically, NK cells can respond rapidly to cellular stress signals and loss of self-recognition, thereby mediating target-cell killing through perforin- and granzyme-dependent cytolysis, death-receptor signalling, and the release of immunomodulatory cytokines. This intrinsic ability to detect and eliminate abnormal cells has made NK cells key mediators of tumour immunosurveillance and an attractive platform for therapeutic development (1). In the field of cell and gene therapy (CGT), NK cells are increasingly explored as an alternative or complement to T cell-based approaches (2). While T-cell therapies have demonstrated major clinical success with Chimeric antigen receptor (CAR) T-cell based methods, their application is still limited by complex manufacturing workflows (3, 4). By contrast, NK-cell-based products offer features that support broader translational use, such as an easier access to allogeneic application allowing for more standardized manufacturing. As a result, NK-cell-based advanced therapy medicinal products are being developed across a rapidly expanding range of clinical settings (5).

As part of the advanced therapy medicinal products, CGTs require robust analytical methods to assess safety, quality and efficacy (6). Among the critical quality attributes (CQAs) of NK-cell products, cytotoxicity is one of the most functionally relevant because it directly reflects the potential anti-tumor activity (7, 8). However, standardization of cytotoxicity testing remains challenging. Biological variability, assay format, analytical workflow, and the absence of broadly harmonized reference standards all influence the magnitude and interpretation of results. Consequently, even when different methods nominally assess the same attribute, they may not deliver directly comparable readouts. A broad range of assays has been used to quantify NK-cell cytotoxicity, most commonly comparing target-cell controls with NK-target co-cultures at defined effector-to-target ratios (E:T), incubation times and interleukin stimulation. Historically, methods included Chromium-51 (9, 10), Europium-TDA (11, 12), lactate dehydrogenase (LDH) (13), and calcein-based release assays (14, 15). More recent studies also employ flow cytometry (FCM)-based single-cell readouts (10, 16, 17), live-cell imaging approaches (18), impedance-based real-time platforms (19), and reporter-based assays (20). Furthermore, functional surrogate assays, such as CD107a degranulation, granzyme delivery and cytokine release, are frequently used to complement these cytotoxicity testing methods, although the readouts do not quantify target-cell killing in the same way as direct cytotoxicity assays (8, 21). Current literature differs substantially in assay principle, incubation time, stimulation, E:T ratio, and reported cytotoxicity range (**Table 1**).

**Table 1:** Cytotoxicity assessment with various methods and corresponding cultivation criteria. Isotope labelled chromium (Cr^51^), europium-thiodiacetat (TDA), lactate dehydrogenase (LDH), calcein-acetoxymethyl ester (AM), flow cytometry (FCM), interleukin (IL).

Method	Source	E:T	Duration	Stimulation	Cytotoxicity range
Cr51	(9)	5:1 – 40:1	4-6h	None	≥20%
	(10)	1:1 – 10:1	4h	None	~20 – 80%
Europium-TDA	(11)	1:1 – 8:1	4h	None	40 – 80%
	(12)	2.5:1 – 20:1	2h	IL2	≤~80% (specific release)
LDH	(13)	10:1	4h	None	20 – 40%
Calcein-AM	(14)	1.25:1 – 10:1	4h	None	30 – 80%
	(15)	1:1 & 2:1	4-8h	None	~20 – 90%
FCM	(10)	1:1 – 10:1	4h	None	~20 – 80%
	(16)	1:1 & 10:1	4h	None & IL2	30 – 60% & 60 – 90%
	(17)	10:1 - 40:1	4h/16 - 22h	None/IL2, IL18, IL21	≤~70%
	(19)	1:1	20h	None	40 – 80%
Live-cell imaging	(18)	1:1 – 5:1	6-24h	None	≤~70%
Impedance	(19)	1:3 – 10:1	24h	None	40 – 80%
Reporter	(20)	0.5:1 – 5:1	6h	None	~50 – 90%

This heterogeneity within cytotoxicity assessment complicates cross-study comparison, and also highlights a broader methodological problem: cytotoxicity measurements are strongly shaped not only by biology, but also by assay design and readout strategy. Even though bulk release methods have been refined and are still commonly used, they often suffer from important limitations: background leakage, spontaneous releases depending on cell density and cross-signal from effector cells, which therefore result in large inter-assay variability and biased cytotoxicity assessments (22). FCM-based cytotoxicity assays address several of these limitations by enabling single-cell analysis of heterogeneous cultures. In addition, FCM allows direct discrimination of effector and target populations and provides more detailed information about target-cell death states, including apoptotic and necrotic phenotypes (23, 24). These features make FCM a particularly attractive platform for standardized cytotoxicity analysis. However, its analytical advantage is partly offset by the continued dependence on manual gating, which introduces operator-dependent subjectivity and limits reproducibility between evaluators. To address this problem, objective and computationally supported analysis strategies are becoming increasingly important in modern FCM (25). Automated gating approaches offer the possibility of reducing evaluator-dependent variability, improving standardization, and enabling reproducible large-scale analysis (26, 27).

In this work, we compared calcein release, LDH release, and a FCM-based assay for the determination of NK-cell cytotoxicity using NK-92 effector cells and K562 target cells. Based on this comparison, FCM was selected as the most suitable platform for standardized evaluation, and an unsupervised autogating workflow was developed for objective analysis of the FCM data. The workflow was benchmarked against three independent manual evaluations across multiple cultivation datasets to determine whether automated analysis could preserve agreement with manual gating while reducing evaluator dependence and improving the standardization of cytotoxicity assessment.

## 2 Materials and methods

### 2.1 Cultures

NK-92 (DSMZ; Goettingen, Germany) and K562 cells, were purchased from the American Type Culture Collection (ATCC; Manassas, U.S.). NK-92 cells were seeded in triplicates into vented T-175 flasks (Thermo Fisher Scientific; Massachusetts, U.S) at 1.0 x 10^5^ cells/mL in 60mL serum-free stem cell growth medium (SCGM; CellGenix, Freiburg, Germany) supplemented with 5% heat inactivated fetal bovine serum (HI FBS; ATCC, Manassas, U.S.) and 2.5 mM GlutaMAX (Thermo Fisher Scientific; Massachusetts, U.S.). K562 cells were seeded in triplicates into vented T175 flasks at 2.0 x 10^5^ cells/mL in 10 mL iscove’s modified dulbecco’s medium (IMDM; Thermo Fisher Scientific Massachusetts, U.S.) supplemented with 10% HI FBS, and 2.0 mM GlutaMAX. K562 cells were split every second day at a ratio of 1:5. Cells were used for cytotoxicity assays, at E:T ratios of 1:1 and 1:5, while their supernatant was used for initial LDH activity assessment. To maintain cell culture volumes, the flasks were refilled with an adequate medium volume to the gain/reach the original volume. Furthermore, the NK-92 cultures were supplemented with a premium grade IL-2 (Miltenyi Biotec; Bergisch Gladbach, Germany) every second day to the final concentration of 500 IU/mL. Cells were grown at standard cultivation conditions, i.e. 37 °C and 5% CO_2_ in a humidified atmosphere. The experiment was conducted for 20 days.

For the automated gating evaluation cytotoxicity data was acquired from a batch, fed-batch and repetitive-batch cultures obtained within optimized and reference medium, in biological and technical triplicates (28). Prior to the start of the experiment, NK-92 cells were adapted to: reference medium alpha-minimum essential medium (MEM; Thermo Fisher Scientific; Massachusetts, U.S.), supplemented with 12.5% HI FBS (Thermo Fisher Scientific; Massachusetts, U.S.), 12.5% horse serum (PAN Biotech; Regensburg, Germany), 24 µM β-Mercaptoethanol (Sigma-Aldrich; Missouri, U.S.), 1x Insulin-Transferrin-Selenium supplements (Gibco, Grand Island, NY, United States) 17.21 μM, 0.69 μM and 0.39 μM, respectively, 2,2 g/L sodium bicarbonate, 0.55 mM L-Arginine (Carl Roth; Karlsruhe, Germany), 0.31 mM L-Glutamine (Carl Roth; Karlsruhe, Germany), 0.05 mM L-Serine (Thermo Fisher Scientific; Massachusetts, U.S.), 0.18 mM myo-Inositol (Sigma-Aldrich; Missouri, U.S.) and 17.64 mM D-Glucose (Carl Roth; Karlsruhe, Germany). Total culture time for each condition was eight days, with daily measurements.

### 2.2 Calcein and LDH assay

2.0 x 10^6^ K562 cells were resuspended in 200 µL containing 15 µM calcein AM green (Invitrogen; Massachusetts, U.S.) in serum-free SCGM medium. The cells were incubated for 30 min at 37 °C in the dark, washed twice with 1x Phosphate buffer saline (PBS; Gibco, Paisley, UK) and resuspended in 3.8 mL serum-free IMDM medium. Afterwards NK-92 cells were harvested and used in E:T ratios 1:1 and 5:1, together with K562 cells. Cocultures were set up by combining 115 µL of both effector and target suspensions to maintain consistent 50/50 (v/v) mixture of both media. The cell suspensions were transferred to a 96-well V-bottom plate (Thermo Fisher Scientific; Massachusetts, U.S) and subsequently incubated for 3.25 h at standard cultivating conditions. Three K562 wells were spiked with 9.2 µL of 5% Triton-X (Sigma-Aldrich; Missouri, U.S) and 9,2 µL of 1x PBS (v/v). Incubation was continued for additional 0,75 h. The 96-well V-bottom plate was then centrifuged at 300 x g for 5 min and supernatants were transferred to a new 96-well V-bottom plate. Centrifugation was repeated one more time to ensure complete removal of cells from the supernatant. 150 µL of each supernatant was transferred to a new 96-flat bottom plate (Thermo Fisher Scientific; Massachusetts, U.S) and analyzed using Tecan Spark microplate reader (Thermo Fisher Scientific; Massachusetts, U.S.). Calcein green was excited at 485 nm and fluorescence detected at 520 nm. Calcein fluorescence-based cytotoxicity was calculated using Equation 1.

Equation 1 Calcein based cytotoxicity. Determination of cytotoxicity from calcein fluorescence, based on relative release of calcein from target cells during coculture. F_coculture_, coculture fluorescence; F_K562_, K562 control fluorescence; F_K562,lysed_, K562 control lysed total fluorescence.


$$Cytotoxicity_{Calcein}\left[\%\right]=\frac{F_{coculture}-F_{K562}}{F_{K652,lysed}-F_{K562}}*100$$

LDH activity was measured using the same supernatants as in the Calcein method. 150 µL of supernatants were transferred into 1.5 mL tubes and quantified using a Cedex Bio HT instrument (Roche; Basel, Switzerland). LDH based cytotoxicity was calculated using Equation 2 (29, 30).

Equation 2 LDH based cytotoxicity. Determination of cytotoxicity from LDH, based on relative release of LDH from target cells during coculture. LDH_coculture_, released LDH in coculture; LDH_NK92_, released LDH in NK92 control; LDH_K562_, released LDH in K562 control; LDH_K562,lysed_, released LDH from lysed K562 control.


$$Cytotoxicity_{LDH}\left[\%\right]=\frac{LDH_{coculture}-LDH_{NK92}-LDH_{K562}}{LDH_{K562,lysed}-LDH_{K562}}*100$$

### 2.3 FCM method

Sampled cells were resuspended in their respective serum free media and transferred to 96-well V-bottom plate, with 2 x 10^5^ cells in total. Cocultures were cultivated in technical triplicates in respective ratios listed below, with additional reference cultures of NK-92 cells and K562 cells alone, each within a final volume of 200 µL. The plate was spun down for 1 min at 100 x g and incubated at 37 °C for 4 h at standard cultivation conditions. Afterwards cells were centrifuged for 5 min at 300 x g, their supernatant was removed, cells were washed with 150 µL FCM buffer (28) and centrifuged again for 5 min at 300 x g.

An E:T ratio of 1:1 and 5:1 was used (v/v) in the method comparison experiments. As K562 cells were used as well, subsequent separation between effector and target cells was performed using the CD56 - PE-cF594 fluorescent labelled antibody (BD Biosciences; New Jersey, U.S.). Furthermore, cell integrity and live/dead determination were performed using Alexa fluor-488–labelled antibody against phosphatidylserine (PS; Merck Millipore, Burlington, U.S.) and pacific-blue amine-reactive dye (AAT Bioquest; Sunnyvale, U.S.), respectively.

For the autogating evaluation, data was acquired using only E:T ratios of 1:1. By employing K562-GFP cells (ATCC; Manassas, U.S.), the CD56 labelling to distinguish effector from target cells was not needed. Live/dead and cell integrity stainings were performed using the DNA intercalating dye 7-aminoactinomycin D (7-AAD; Thermo Fisher Scientific; Massachusetts, U.S) and Annexin V-BUV421 (BD Biosciences; New Jersey, U.S.) against PS.

**Figure 1:**
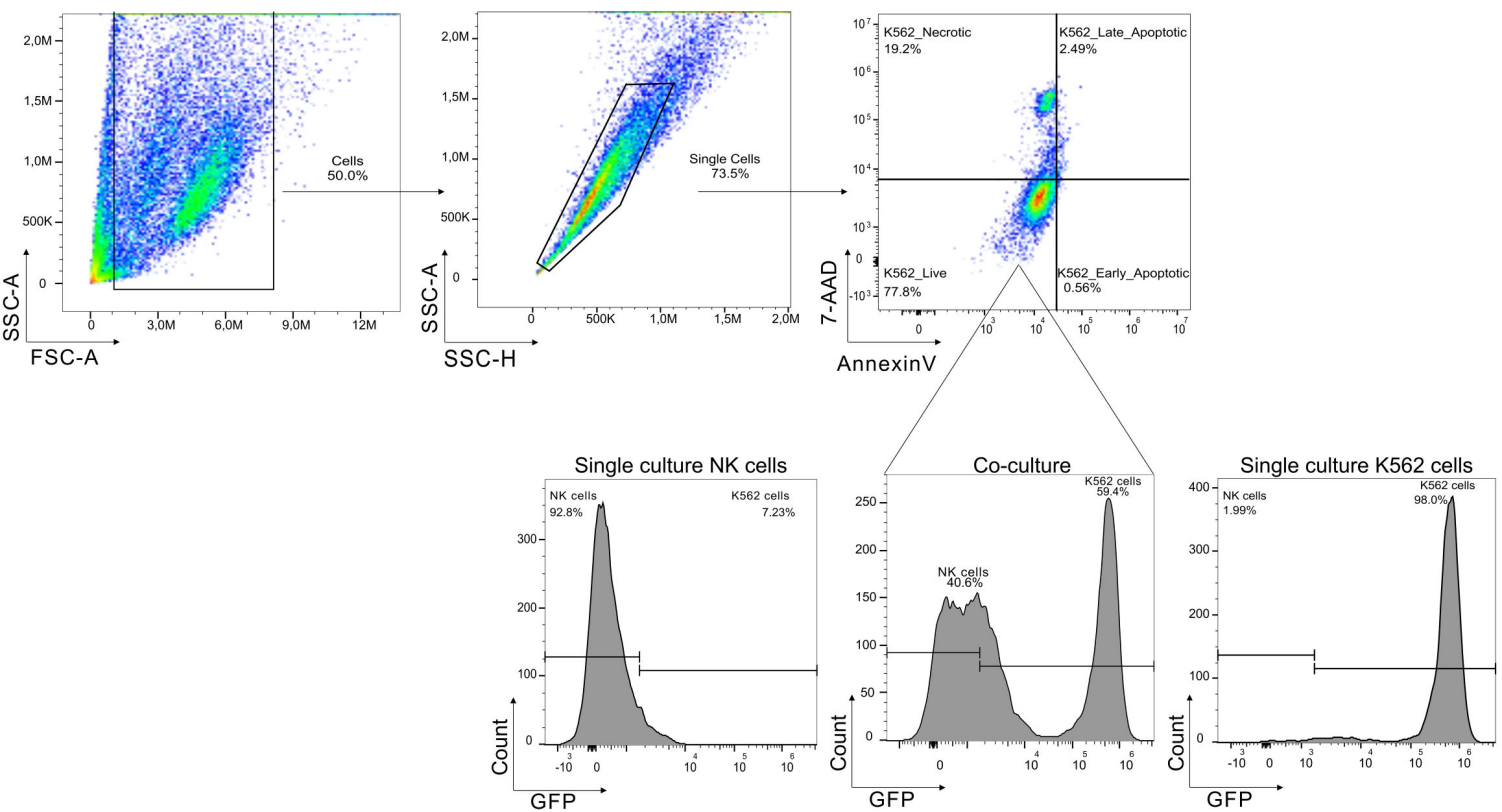
Manual gating strategy for cytotoxicity assessment. The gaiting strategy (top left to right): remove cell debris based on size beads in FSC-A and SSC-A gate, select single cells in SSC-H and SSC-A gate and define quadrants in Annexin V and 7-AAD gate. The quadrants were selected using K652 and NK-92 cells, derived from GFP based gate of single cultures (bottom left and right), with the gating transferred to GFP signal in the coculture (bottom middle).

Briefly, the washed cells were resuspended in 100 µL AnnexinV-BV421 stock (0.625 µL AnnexinV-BUV421 (BD Biosciences; New Jersy, U.S.) in 100 µL/well 1x annexin binding buffer (ABB; Thermo Fisher Scientific; Massachusetts, U.S.) and transferred to a 96-well V-bottom plate. Following a 15 min incubation at RT in the dark, 150 µL/well 1x ABB was added, the plate was centrifuged for 5 min at 300 x g and the supernatants removed. Cells were resuspended in 200 µL/well 1xABB or 50 µL 7-AAD (5 µg/mL in 1xABB) respectively. Each plate also included size calibration beads (Thermo Fisher Scientific; Massachusetts, U.S.), used to set the cutoff between debris and cells. FCM analysis was performed using CytoFlex S (Beckman Coulter; Brea, U.S.). The manual gating strategy for cytotoxicity evaluation is shown in **Figure 1**.

The first gate forward scatter – area against side scatter - area (FSC-A/SSC-A) was used to exclude debris at a cutoff < 8 µm from cells (31), determined using size beads. Subsequently in the second gate side scatter – height (SSC-H) against SSC-A doublets events were excluded. In the final gate (7-AAD/AnnexinV), 7-AAD and AnnexinV were used as markers for live/dead and apoptosis determination, respectively. The vertical and horizontal gates were transferred from the single cultures, differentiating NK-92 cells (GFP^-^) and K562 cells (GFP^+^). Cytotoxicity was assessed by calculating a percentage of total events in their respective quadrants from coculture data and target cell data as shown in Equation 3 (32).

Equation 3 Flow cytometry-based cytotoxicity calculation. Determination of cytotoxicity from flow cytometry gates, based on K562_live_ [%]; percentage of live K562 cells in reference medium and $Coculture_{K562_{live}}\left[\%\right]$; percentage of live K562 cells in coculture medium. Percentages calculated relative to total events in the respective gate.


$$Cytotoxicity_{FACS}\left[\%\right]=\frac{K562_{live}\left[\%\right]-Coculture_{K562_{live}}\left[\%\right]}{K562_{live}\left[\%\right]}*100$$

### 2.4 Manual gating evaluation

The influence of subjective manual gating contribution to measurement variability was calculated using a linear mixed-effects model. The model was fitted to cytotoxicity values obtained by three independent users (i.e. researchers) using the restricted maximum likelihood estimation (33). Each user gated the same set of FCM files obtained across nine experimental days, two media conditions and three culture modes (28). Prior to model fitting, groups with cytotoxicity ≤ 5% or ≥ 25 CV% were excluded to focus on biologically relevant measurements. Media conditions and culture modes were set as fixed effects, day as a random intercept, and users as an additional variance component (Equation 4).

Equation 4 Variance decomposition of cytotoxicity. (β_0_), intercept; (β_1_Media condition_i_), fixed effect for media condition; (β_2_Culture mode_j_), fixed effect for culture mode; (b_Day,k_), random intercept for day; (b_Useres,l_), random intercept for users; (ϵ_ijkl_), residual error.


$$\begin{array}{l} Cytotoxicity_{ijkl}=\beta_{0}+\beta_{1}Media\;condition_{i}+\beta_{2}Culture\;mode_{j}+b_{Day,k}+b_{Useres,l}+\epsilon_{ijkl}\\ where\;b_{Day,k}\;∼N\left(0,\sigma_{Day}^{2}\right),\;b_{Users,l}\;∼N\left(0,\sigma_{Users}^{2}\right)\;and\;\epsilon_{ijkl}\;∼N\left(0,\sigma_{\epsilon }^{2}\right), \end{array}$$

Model comparison between the full model and a reduced model (without the user variance component) was performed and evaluated using likelihood-ratio testing, and information criteria (**Supplementary Appendix 1**).

### 2.5 Automated gating pipeline

The evaluator-dependent variability in the described FCM-based cytotoxicity assessment was reduced by implementing a custom Python-based autogating pipeline for standardized analysis of NK-cell cytotoxicity against K562 target cells. The pipeline was designed to reproduce the core logic of the manual assay readout while minimizing subjective gate placement and improving reproducibility across experimental runs. For each experimental unit, the pipeline analyzed the corresponding effector-only control, target-only control, and coculture files together. Total events were first subjected to an initial rectangular gate in forward- and side-scatter space to remove low-scatter events, in correspondence with the bead-based cut-off within the manual gate and acquisition noise. In the optimized workflow, the lower FSC-A boundary was fixed at the value of 2.4 × 10^6^, equivalent to< 8 µm, while SSC-A was retained within the recorded range. The FSC-A threshold of 2.4 x106 is specific to the CytoFLEX S instrument (Beckman Coulter) used in this study and was calibrated using size-reference beads corresponding to an 8 µm cut-off. For other flow cytometry platforms, users should determine equivalent FSC-A thresholds through instrument specific bead calibration. A second morphology-cleaning step was then applied in SSC-H versus SSC-A space using a custom robust ratio-based filter, thereby enriching singlet-like events and reducing the influence of aggregates and morphologically atypical events. Effector and target populations were subsequently separated using the target-associated fluorescence channel using the green fluorescent protein signal. To identify the mixed-population structure in cocultures, kernel density estimation was applied to the fluorescence distribution. For operational gating of the single-culture controls, the separation threshold was derived from the effector-only control as the right boundary of the principal effector peak. Events below this threshold were retained as effector cells and events above the threshold as target cells. This step provided a standardized rule for effector-target discrimination while preserving sample-specific adaptation. Cytotoxicity was quantified in the Annexin V versus 7-AAD space using gated effector-only and target-only controls as reference populations. In the optimized workflow reference events were selected using a 2-dimensional k-nearest-neighbor density approach based on Euclidean distance from the densest point, retaining 95% of events in each reference distribution. Quadrant thresholds were then derived separately for the effector and target reference populations using the 90th percentile of each marker axis. These identified thresholds were applied to the coculture-derived target population to quantify viable, early apoptotic, late apoptotic, and necrotic target-cell fractions. Cytotoxicity values were calculated using the same formula as in manual gating (Equation 3). All metrics used for optimization and further explanation for each method is provided in **Supplementary Appendix 2**.

### 2.6 Method evaluation and comparison with manual gating

In the method comparison experiment, differences have been evaluated for time and method parameters using a two-way ANOVA. To determine the precision between the methods, one-way ANOVA was employed. Significance was assessed at p< 0.05. For method selection and improvement, precision was used to determine the best performing method (Equation 5). Precision refers to one measurement of technical triplicates. If biological triplicates were measured, the average of the determined precision was used.

Equation 5 Precision calculation CV%. Method precision determination of cytotoxicity based on Std(), standard deviation; and Mean(), mean; of cytotoxicity from technical triplicates.


$$CV\%\left[\%\right]=\frac{Std\left(Cytotoxicity\right)}{Mean\left(Cytotoxicity\right)}*100$$

In case of the manual evaluation, CV% was first computed separately for each user and then averaged across users, while for the automated pipeline, CV% was computed from the corresponding automated evaluation. The association between cytotoxicity and CV% advantage was assessed using Spearman correlation. An exploratory breakpoint analysis scanned cytotoxicity thresholds from 10% to 75% in 5% increments to identify the approximate range above which autogating showed a significant precision advantage. At each threshold, paired differences were evaluated using Wilcoxon signed-rank tests.

A matched comparison subset (n = 19) was defined as a set of biological-replicate groups, for which technical triplicates were available, all three users and the autogating pipeline returned a value, and the group passed the pre-analysis filter (≥ 5% cytotoxicity, CV% ≤ 25%). This subset was used to assess agreement between autogating and pooled manual evaluation using Pearson correlation, Lin’s concordance correlation coefficient, mean absolute error, root mean square error and median absolute difference. Furthermore, inter-rater consistency was quantified using interclass correlation coefficients ICC(3,1), a two-way mixed-effects, single-measure, consistency intraclass correlation coefficient (34). The analysis was performed first across the three manual users and then after including autogating as a fourth rater. Finally, mean bias for autogating and for each user was assessed using a leave-one-out setup using Bland-Altman approach, in which each evaluator was compared against the pooled mean of the remaining three evaluators (35).

### 2.7 Software environment

All analyses were performed on a 64-bit Windows system using Python 3.10.20. The environment specifications were comprised of: numpy 1.26.4, pandas 2.3.3, scipy 1.15.2, scikit-learn 1.7.2, matplotlib 3.10.8, seaborn 0.13.2, openpyxl 3.1.5, FlowCytometryTools 0.5.1, and statsmodels 0.14.6. FCS files were imported with FlowCytometryTools, numerical processing and statistical analyses were performed with numpy, pandas, scipy, and statsmodels, machine-learning based gating utilities used scikit-learn, and visualizations were generated with matplotlib and seaborn.

## 3 Results

### 3.1 Method comparison

**Figure 2:**
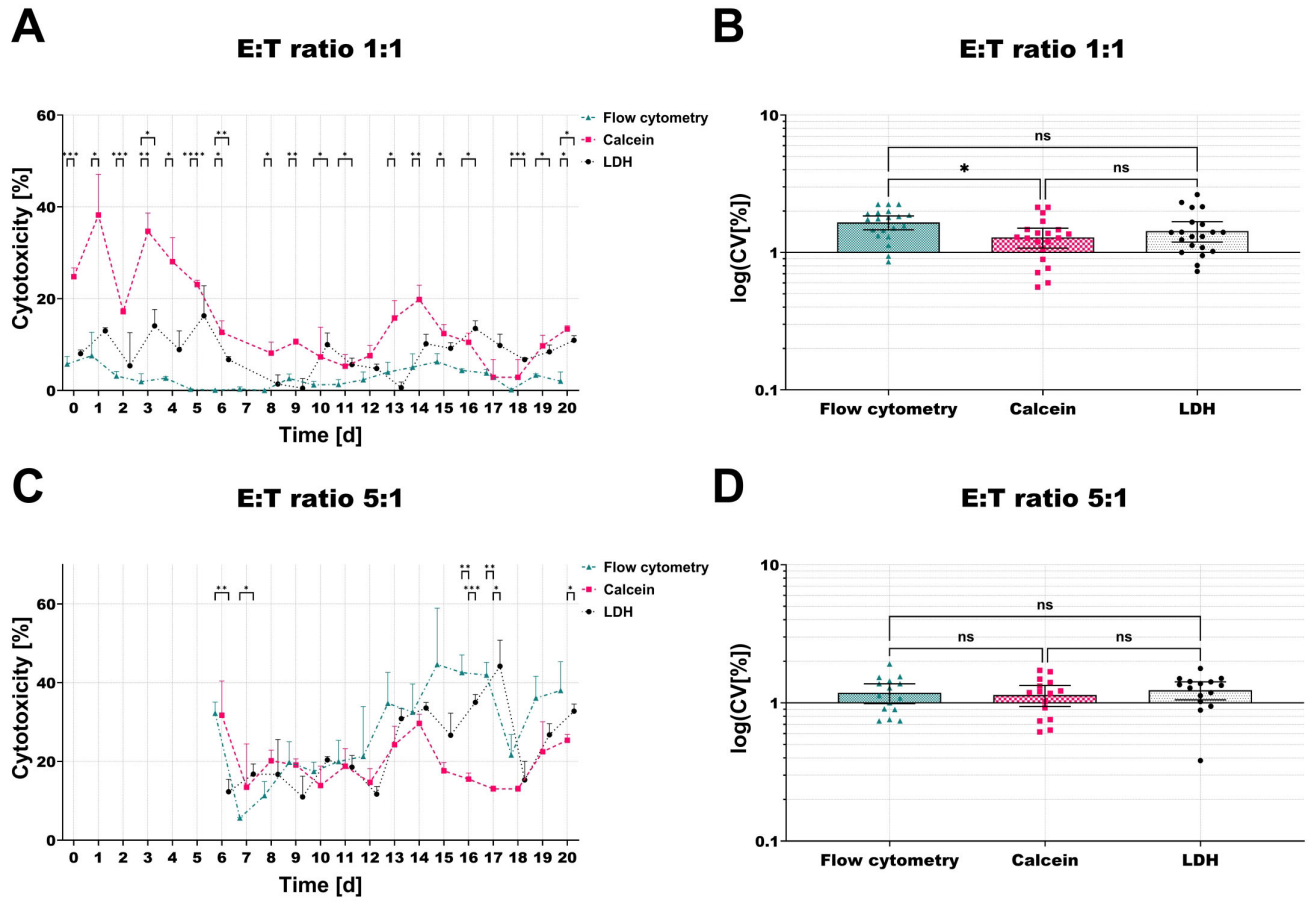
Cytotoxicity and precision comparison between methods. Cytotoxicity over time using E:T ratios 1:1 **(A)** and 5:1 **(C)**, were evaluated using flow cytometry (green), Calcein (red) and LDH (black) methods, displayed with standard deviations for each measurement (n=3). The minimum cell requirements for cytotoxicity assay in the E:T ratio 5:1 were reached on day 6. Statistical analysis was performed for each timepoint and method using two-way ANOVA of time resolved data **(B, C)**. One-way ANOVA **(B)** and mixed-effects analysis **(D)** were performed on log transformed precision values of observed data log(CV[%]). Significance is depicted via: *p< 0.05, **p< 0.01, ***p< 0.001, ****p< 0.0001; ns, not significant.

In an *in vitro* co-culture experiment of NK-92 and K562 cells, we assessed the cytotoxicity of the NK cells by applying three commonly used assays: FCM, calcein release and LDH release. Cytotoxicity assessment was conducted using all three methods at E:T ratios of 1:1 and 5:1, for every timepoint during culture (**Figures 2A, C**). Significant differences between methods were observed at multiple time points, indicating that the measured magnitude of NK-92-mediated cytotoxicity was strongly dependent on the used method. Assay precision analysis across all three tested methods revealed differences between FCM and calcein based assays in the 1:1 ratio group. However, within the 5:1 ratio group, we could not observe differences (**Figures 2B, D**).

At an E:T ratio of 5:1, the three methods yielded more comparable cytotoxicity values with only six significantly different measurements. Compared to E:T 1:1, most measurements were significantly different in at least one method, totaling 21 significant differences. With regards to precision no significant differences were observed at an E:T ratio of 5:1. However, the 5:1 condition required substantially higher effector-cell numbers, which prevented cytotoxicity assessment during the first five days of culture. As a result, the higher E:T condition was less suitable for early-stage monitoring of culture performance despite its stronger overall killing signal. Furthermore, large variations in cytotoxicity levels were observed over culture time, with deltas of up to 35% and 30% in E:T ratios of 1:1 and 5:1 respectively (**Figures 2A, C**). This indicates that NK-cell cytotoxicity was neither constant nor consistent over the assay method and culture duration. For method comparison, only measurements fulfilling a minimum acceptance criterion of CV% less than or equal to 25% were included. A significantly better precision of calcein compared to the FCM-based method was observed at an E:T ratio of 1:1, which was not present at an E:T ratio of 5:1 (**Figures 2B, D**). Although the calcein release assay demonstrated a better precision at an E:T ratio of 1:1, assay precision was not the sole criterion considered in the decision to move forward with FCM based methodology. We believe that unlike release-based assays, FCM provides a single-cell resolution with simultaneous discrimination of effector and target populations that overrules the higher precision of the calcein-release assay at a lower E:T ratio. We therefore chose to improve the reproducibility and standardization of an FCM-based approach.

### 3.2 FCM method refinement

**Figure 3:**
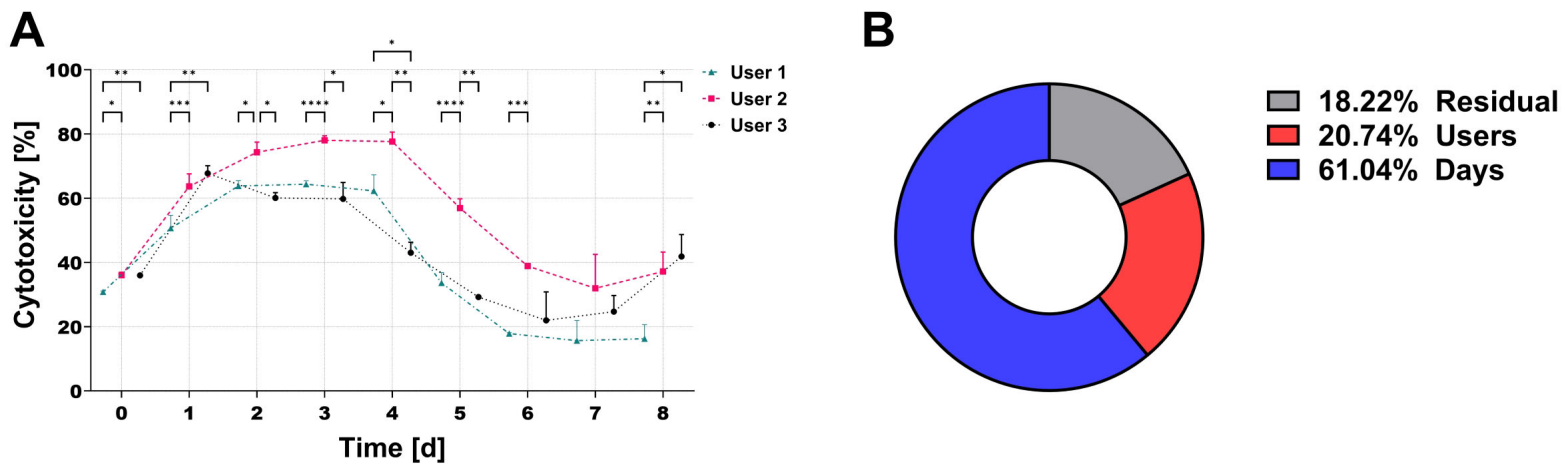
Cytotoxicity evolution over time of one trajectory and variance decomposition of cytotoxicity from manual gating. **(A)** Flow cytometry-based cytotoxicity (n=3) evaluated by three independent users. One-way ANOVA was performed between groups on each discrete timepoint. Significance is depicted as: *p< 0.05, **p< 0.01, ***p< 0.001, ****p< 0.0001. **(B)** Linear mixed-effects model fitted to partition the total variance within the complete dataset, depicting the percentual total variance of measurement day, residual and the manual users.

To refine the FCM-based cytotoxicity assay, a second dataset with increased experimental complexity was analyzed. This dataset included three cultivation methods, two media conditions, three biological replicates, and eight cultivation days, resulting in 144 unique cytotoxicity measurements, each assessed in technical triplicates. Manual analysis required sequential placement of three user-defined gates before final cytotoxicity was calculated. Differences in cytotoxicity determination among the three users are shown over time, with significant differences marked among the users (**Figure 3A**). The overall trajectory of cytotoxicity was preserved across users, while the magnitude of the reported cytotoxicity values differed significantly at multiple time points, by up to 20%. To further quantify this effect across the full dataset, variance decomposition was performed to estimate the contribution of user-dependent variability to total measurement variance (**Figure 3B**). This analysis showed that user-dependent gate placement contributed 20.74% to the overall variability of the assay, alongside biological and residual sources of variation.

### 3.3 Automated gating

**Figure 4:**
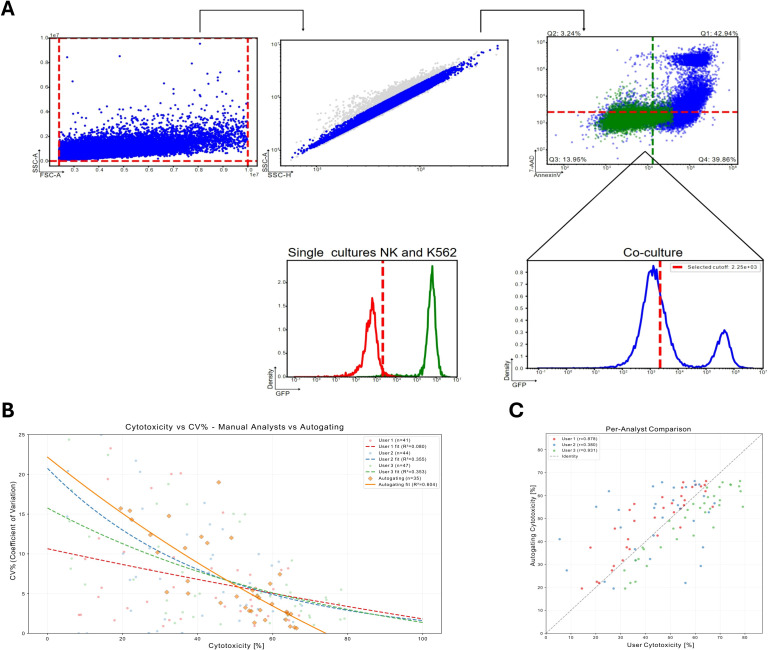
Auto gating strategy for cytotoxicity assessment and overall precision (CV%) of evaluated cytotoxicities. Analogue to the manual gating strategy, **(A)** the auto gating strategy (top left to right): remove cell debris based on size beads; select single cells; quadrants based on K652 live cells (green) overlapped with the coculture results. The single cultures of K652 cells (green) and NK-92 (red) were used to define the cutoff within the coculture (bottom left to right). **(B)** Cytotoxicity over precision, cutoff at ≤ 5% cytotoxicity and ≥ 25% CV%, remaining points (n) were used to fit exponential curves for each user and the automatic gating approach with coefficients of determination (R²). **(C)** Users against autogating predictions of cytotoxicity determined values, wit Pearson correlation coefficient for each user.

An automated gating (autogating) pipeline was developed to perform cytotoxicity evaluation without user interaction using unsupervised analysis steps. The autogating pipeline follows the same steps as the manual evaluation (A). A pre-analysis filter of ≤ 5% cytotoxicity and ≥ 25% CV% was applied to exclude near-background measurements and poorly precise measurement replicates. Exponential fits were used to indicate the trend of how precision improves as cytotoxicity increases for users and autogating (**Figure 4B**). According to breakpoint analysis, autogating showed a relative increase in precision compared to manual gating, with the crossover point occurring at approximately 45% cytotoxicity. When comparing autogating to the different manual user evaluations, the overall Pearson correlation coefficients indicate a good performance of the pipeline (**Figure 4C**).

Furthermore, autogating was evaluated relative to manual gating. The pooled manual evaluation was used as a reference standard representing the consensus of manual assessments. Across a matched subset of 19 comparison groups, autogating showed strong concordance with manual analysis: an overall Pearson correlation coefficient of r = 0.7667, and Lin’s concordance correlation coefficient of 0.8810. Absolute error remained limited, with a mean absolute error of 3.55%, a root mean square error of 4.54%, and a median absolute difference of 2.49%. Inter-rater consistency among the three manual users was moderate, with ICC (3,1) = 0.497, increasing to ICC (3,1) = 0.603 when autogating was included as a fourth rater. Using the leave-one-out Bland-Altman approach, autogating showed the smallest absolute mean bias (-0.45%). By contrast, the manual users showed larger evaluator-specific biases, with mean differences of - 5.67% for user 1, - 6.25% for user 2, and + 12.37% for user 3. To identify the best-performing configuration, a grid search across candidate pipeline combinations was performed and the top-ranked workflow was selected for downstream evaluation (**Supplementary Appendix 2**). Example datasets and code are available on GitHub (https://github.com/SzAleks/AutoCytotox).

## 4 Discussion

In the initial experiments we demonstrated that commonly used methods for cytotoxicity evaluation led to inconsistent results. This observation is consistent with prior reports that cytotoxicity readouts are strongly influenced by assay design. In particular, calcein and LDH release assays are susceptible to background signal arising from spontaneous cell death and do not directly distinguish effector-derived from target-derived signals (22). Although standard cytotoxicity calculations include correction terms based on single-culture controls, these corrections cannot fully eliminate signal accumulation caused by ongoing spontaneous apoptosis or nonspecific release. By contrast, FCM enables direct target-cell identification and apoptosis-state classification at the single-cell level, providing a more specific readout of target-cell death (23, 24).

Cytotoxicity assays are routinely performed at multiple E:T ratios (**Table 1**), with increasing amounts of effector cells, resulting in more target killing. We chose to use the method only at an E:T ratio of 1:1 for further FCM method improvement. This ratio requires fewer effector cells, providing early-stage measurements and therefore enabling early insight into the culture performance. More importantly, high E:T ratios (10:1 or higher) are rarely encountered *in vivo* and may overstate the NK potency. For most tumors, target cells (here cancer cells) vastly outnumber the therapeutic dose of effector cells. In these circumstances, effective killing occurs at comparatively low E:T ratios. Thus, assays at E:T ratios of 1:1 are more representative for their cytotoxic effect on cancer cells. As we already observed relatively low cytotoxicity at E:T 1:1 after a 4 h assay, we decided to set these conditions as our minimal requirements, to ensure that our measurements reflect actual balances of effector to target cells in clinical settings (36). It should be noted that the protocol was established using cell-line-based effector and target cells. Its application to primary NK cells or CAR-NK cells may require assay-specific adaptation.

It is worth mentioning that cytotoxicity should not be regarded as a constant, but rather a dynamic functional readout that depends on assay duration, culture state, and experimental design, as it can be also observed in our results. Cytotoxicity readout of NK-cells can be affected by different cytokines (12, 15–17, 21) or restored by culture conditions (37). Therefore, measurements obtained at determined time points may provide only a partial representation of the true NK cytotoxicity. This makes the cytotoxicity assessment not only an analytical problem, but also a process-development problem, in which time-resolved and methodologically standardized evaluation becomes critical for meaningful comparison across experimental conditions.

In order to improve the FCM-based cytotoxicity evaluation, manual evaluations were performed, acting as a baseline for the automated workflow development. Human evaluation is prone to subjectivity, while the variability introduced during gating can accumulate and contribute to overall assay error. To assess the magnitude of this effect, the full dataset was independently analyzed by three users. All users reproduced the general temporal trends in cytotoxicity, indicating that the assay captured consistent biological dynamics across culture time. However, the absolute cytotoxicity values assigned to the same samples differed substantially between users. Using variance decomposition, 20.7% of total variability was attributed to the users alone. This inter-operator assessment should be interpreted in light of the study design, as analysis was based on three users and 19 matched samples, which may not fully capture the diversity of gating strategies encountered in broader practice. Therefore, the quantitative estimate of user-dependent variability should be considered assay- and dataset-specific. Future studies including additional operators, larger datasets, and independent laboratories are required to further assess the generalizability of this effect.

Even with this newly established autogating workflow, biological and technical triplicates resulted in a few highly noisy measurements. According to the observed trends (**Figure 4B**), cytotoxicity results from users and autogating are subjects to overall decreasing precision at low cytotoxicity. As it cannot be distinguished if target cells are actively lysed by effector cells or die due to apoptosis at low cytotoxicity values, we defined a lower limit of cytotoxicity determination at ≤ 5% for the evaluation of autogating evaluation, much lower than what is usually required for this CQA (38). Even though samples were processed in biological triplicates and analyzed in technical triplicates, some measurements resulted in poor precision. A precision cutoff at ≥ 25% was applied to exclude poorly precise measurement replicates, prior to any follow-up analysis (39, 40). The variance decomposition was performed after excluding very low cytotoxicity values (≤ 5%), which were reported to have reduced quantitative reliability (41) and high-variability observations (CV% ≥ 25%) (39) to ensure robust model fitting within a biologically interpretable measurement range. However, these exclusion criteria may limit generalizability of the estimated variance components, as excluded conditions consisted of extreme low-signal or high-variability observations, where manual gating variability is typically more pronounced. As a result, the reported contribution of manual gating (20.74%) reflects variability within a biologically reliable measurement range and may represent a conservative estimate of overall user-dependent bias. Our optimized autogating pipeline resulted in a uniform increase in precision across the full analyzed data range. The breakpoint analysis indicated a gradual shift in precision between methods, with autogating showing increasing advantage over manual gating at clinically relevant NK potency levels (around 45%) (38), while remaining comparable across lower cytotoxicity ranges. Importantly, the increased precision observed at higher cytotoxicity was not accompanied by loss of agreement with manual evaluations. On the matched subset, autogating retained strong concordance with pooled manual analysis while maintaining limited absolute error, indicating that the optimized workflow preserved the overall structure of the manual readout. Furthermore, autogating showed the smallest absolute mean bias, compared to each user evaluation. These results present autogating as a workflow that primarily improves standardization and reduces evaluator dependence while preserving strong agreement with reference manual analysis.

In this context, the proposed workflow should be distinguished from established automated or semi-automated flow cytometry analysis frameworks, including FlowSOM (42), flowDensity (43), openCyto (44), and CytoNorm (45, 46). While these tools improve reproducibility through automated population identification, gate placement, template-based gating or data normalization, their primary focus lies in population analysis, harmonization within individual datasets and predefined gating structures. In contrast, the presented workflow integrates multiple annotated datasets into a single cytotoxicity endpoint, thereby reducing variability from manual gate transfer, evaluator-dependent interpretation, and cross-dataset result integration. Thus, the method represents a targeted endpoint-generation workflow for cytotoxicity assays, rather than a general-purpose automated gating framework.

## 5 Conclusion

In this study, we assessed well-known cytotoxicity evaluation approaches and developed an autogating approach for the FCM-based cytotoxicity evaluation. We showed that cytotoxicity estimates strongly depend on the assay format used. Release-based assays and FCM readouts produced differing cytotoxicity values under the same experimental conditions, supporting the need for a more specific and standardized evaluation strategy. On this basis, FCM was selected as the most suitable platform for further assay refinements it enables target-cell-specific, single-cell-resolved assessment of cytotoxicity in GFP-labeled K562 target cells, without the shortcomings of conventional release assays.

We further showed that manual FCM evaluation introduced a substantial source of measurement uncertainty. Although independent users reproduced similar overall cytotoxicity trends, the absolute magnitude of the reported values differed between users, and variance decomposition confirmed that user-dependent processing contributed measurably to total assay variability. These findings identified manual gate placement as an important source of inter-user bias and limited reproducibility.

To address this limitation, we developed an unsupervised automated gating pipeline, analogue to the manual evaluation, for FCM-based cytotoxicity evaluation and optimized it by systematic grid search using a broad experimental dataset set. The final workflow preserved good agreement with manual analysis while eliminating evaluator-dependent gate placement, allowing for reproducible deterministic results. Autogating did not uniformly result in more precise measurements across the entire assay range. Instead, autogating showed cytotoxicity-dependent performance, with a precision advantage at higher cytotoxicity values, while maintaining low overall bias and good concordance with manual reference measurements.

Taken together, these findings support the FCM-based autogating as a standardized, reproducible, and evaluator-independent approach for NK-cell cytotoxicity assessment. We present a flexible automated workflow that is adaptable to similar FCM-based cytotoxicity assays. Its main goal was not only automatization, but the reduction of user-dependent variability and the establishment of a more harmonized analytical framework. This may enable more comparable cytotoxicity measurements across experiments, users, and laboratories and thereby contribute to improved standardization of functional immune-cell assays in both preclinical and translational settings.

## Data Availability

The raw data supporting the conclusions of this article will be made available by the authors, without undue reservation.
