# Supplementary material for: Automated cytotoxicity assessment of natural killer cells by flow cytometry
Source: Front Immunol. 2026 Jun 29;17:1868673. doi: 10.3389/fimmu.2026.1868673 (PMC13357856; doi:10.3389/fimmu.2026.1868673)
Supplement: Supplementary file 1 [file SupplementaryFile1.pdf]

Appendix 1

Contribution of manual gating subjectivity to measurement variability

After filtering with 25% precision cutoff and 5% cytotoxicity, 132 user-based evaluations remained to fit the full model using restricted maximum likelihood. The variance decomposition from the fitted model attributed roughly 61.1% of total variance to day-to-day differences ($\sigma^2 = 253.05$), 20.7% to user-dependent manual gating variability ($\sigma^2 = 85.96$), and the remaining 18.2% to residual error ($\sigma^2 = 75.51$). Residual diagnostics showed only minor deviation from normality with Shapiro–Wilk ($W = 0.968$, $p = 0.035$), with minor tail deviation in the Q-Q plot, which does not meaningfully violate the model's assumptions. Thus, after accounting for media and culture mode, about one-fifth of the variability in manual cytotoxicity measurements was attributable to the users. To evaluate the user effect, the full model was compared by maximum likelihood with a reduced model lacking the user random effect, using likelihood-ratio testing and Akaike information criterion (AIC) (Vaida and Blanchard 2005).

Model comparison strongly supported inclusion of the user component: the full model had a higher log-likelihood (-497.87 vs. -527.40) and lower AIC (1009.74 vs. 1066.81) than the reduced model, with a highly significant likelihood-ratio test ($\chi^2(1)=59.07$, $p<0.001$). Therefore, subjective gating contributes a statistically significant source of variability beyond the fixed experimental factors and day-to-day biological variation.

In summary, the mixed-effects analysis confirms that manual gating introduces a distinct user-dependent variance component. Deterministic autogating therefore not only saves labour but also reduces this source of user-specific variability. Given that cytotoxicity is a bounded percentage, and the model assumes gaussian random effects and residuals, the reported variance percentages should be interpreted as relative contributions within this filtered dataset rather than as universal constants.

Literature

Vaida, Florin, and Suzette Blanchard. 2005. *Conditional Akaike Information for Mixed-Effects Models*. Vol. 92. https://academic.oup.com/biomet/article/92/2/351/233128.
