# Supplementary material for: Automated cytotoxicity assessment of natural killer cells by flow cytometry
Source: Front Immunol. 2026 Jun 29;17:1868673. doi: 10.3389/fimmu.2026.1868673 (PMC13357856; doi:10.3389/fimmu.2026.1868673)
Supplement: Supplementary file 2 [file SupplementaryFile2.pdf]

Appendix 2

Autogating pipeline optimization

Complementary to the already described pipeline of autogating, this appendix documents: (i) the systematic hyperparameter optimization from which the operational defaults were chosen, (ii) the alternative methods that were evaluated.

1. Optimization design and ranking metrics

Hyperparameter selection was performed using a grid search across parameters that influence the final outcome. The optimization evaluated 1323 parameter combinations across all datasets, of which 1301 completed successfully and 1104 yielded a complete set of ranking metrics after application of the pre-analysis cytotoxicity (≥ 5%) and precision (CV ≤ 25%). Candidate workflows were ranked by a composite of complementary scores using mean CV%, Pearson correlation, Spearman rank correlation (Schober and Schwarte 2018), Lin's concordance correlation coefficient (Watson and Petrie 2010), mean absolute error, root mean square error, and mean bias evaluated within the Bland–Altman framework (Gerke 2020). Besides mean CV%, all metrics were evaluated against the pooled mean values from the three users. This multi-metric ranking was used, because no single statistic test alone is sufficient to evaluate the methods performance. To be able to align all metrics, Borda based rank transform procedure was used (Pihur, Datta, and Datta 2009). The scores were calculated by ranking each of these seven metrics from one (best) to 1104 (worst) within all parameter combinations. Pearson correlation, Spearman rank correlation and Lin's concordance correlation coefficient ranking were inverted, as larger values represent the favoured outcome for these metrics. By summing the metric scores, the lowest score of 483 was identified, yielding the optimized generalizing hyperparameter combination. The corresponding metric values are shown in Table 1.

Table 1 Optimized workflow evaluation metrics

Metric	Value
mean CV%	6.617
Pearson correlation	0.8817
Spearman rank correlation	0.7667
Lin's concordance correlation coefficient	0.8810
mean absolute error	3.548 %
root mean square error	4.545 %
mean bias (Bland–Altman)	0.006

2. Autogating pipeline alternative methods

The grid search varied multiple hyperparameters spanning four functionally distinct decision points of the pipeline: the singlet/morphology gate (Gate 2), kernel-density-estimate (KDE) smoothing of the green fluorescence protein (GFP) distribution, the reference-derived quadrant cut-off, and the proximity rule that defined K562-like co-culture events. For each decision point, the alternative methods and chose optimized method are summarised below. Parameter values that were searched but not selected are listed explicitly to document the negative result. The method names applicable through the command line interface of the public GitHub repository.

2.1 Singlet/morphology gate (--gate2_method)

The singlet gate was applied immediately after the bead gate and before any fluorescence-based decision. Three implementations were evaluated: (i) knn: a k-nearest-neighbour (KNN) density threshold was used in the (SSC-H, SSC-A) plane (Biau and Devroye 2015). Since the threshold was calculated per well, therefore depending on the local event count, which caused the threshold to drift between technical replicates of the same biological condition;

(ii) linear_band: robust linear fit $y = ax + b$, with a median ± 3 × median absolute deviation (MAD) acceptance band on the residuals (Leys et al. 2013). In co-culture wells the fit is pulled toward whichever population (NK or K562) dominates the well. This introduces a bias, towards the gate with the minority cell type, typically the surviving K562 in high-killing wells; (iii) ratio (selected) — median ± 3 × MAD acceptance band on log_{10}(FSC-A/FSC-H), produced the lowest within-replicate CV of the three options on this dataset.

2.2 GFP-distribution KDE bandwidth (--bw_adjust)

The Gaussian-KDE bandwidth multiplier was applied to the per-well histogram of GFP, prior to the local-minimum search that defined the GFP^{+}/GFP^{-} threshold and was searched at bw adjust ∈ {0.05, 0.10, 0.20} (Hahne et al. 2009). Larger values over-smoothed the bimodal histogram and merged the GFP^{-} effector peak with the GFP-low debris shoulder. This would later eliminate the local minimum required by the threshold-finding step, forcing the well to be treated as un-gateable. Smaller values under-smoothed and introduced false local minima. The selected bw_adjust = 0.05 was the smallest value that produced a well-defined minimum in every ranking-eligible well.

2.3 Quadrant gate derivation (--gater_derivation)

The Annexin V / 7-AAD quadrant cut-offs that define the viable subset of the K562-only reference were derived in three competing ways: (i) max: uses the maximum value of the reference distribution as the cut-off. It is anchored to the most extreme single event and therefore highly sensitive to outliers; (ii) mean_ksd: uses mean ± σ × standard deviation, with σ ∈ {2.0, 3.0, 4.0}.This method under-cut the viable population at σ = 2.0 and over-cut it at σ = 4.0, with no value of σ outperforming the percentile alternative; (iii) percentile (selected): used the empirical quantile of the reference distribution, with percentile ∈ {0.80, 0.90, 0.99}. It is non-parametric, robust to right-tail outliers, and stable across replicates of the control well. The optimum at percentile = 0.90 was determined as the best setpoint.

2.4 Proximity rule (--method)

The proximity rule is the last gating operation of the pipeline, applied immediately before quadrant counting. It operates on the (Annexin V, 7-AAD) plane and is applied separately to the effector reference (NK-only well) and the target reference (K562-only well), refining each cloud to a high-confidence subset against which the co-culture quadrants will be set. Three different methods were evaluated: (i) axis: estimated a one-dimensional KNN density on the Annexin V axis and on the 7-AAD axis independently, identified the densest point on each axis, and retained 95% of events closest to the densest point on that axis. The two per-axis sets were then intersected, defining a rectangular acceptance region around the densest (Annexin V, 7-AAD) corner. Because each axis was treated independently, the resulting rectangle ignored the diagonal correlation between Annexin V and 7-AAD that arises during early-to-late apoptosis transitions; (ii) density: estimated the local event density in the 2D (Annexin V, 7-AAD) plane via the KNN, sorted events by their density value, and retained the top 95 %. In high-cytotoxicity wells, the surviving K562 reference became too sparse to support a stable density estimate, producing unreliable acceptance boundaries; (iii) euclidean (selected): estimated a two-dimensional KNN density to identify the single densest (Annexin V, 7-AAD) point, then retained 95% of events with the smallest Euclidean distance to that point. This method was selected as the best one during optimization.

2.5 Proximity quantile (--percent)

The retained-fraction parameter of the previous proximity rule was searched at percent ∈ {0.65, 0.70, 0.75, 0.80, 0.85, 0.90, 0.95}. The same value was applied independently to the effector reference (NK-only well) and the target reference (K562-only well). Lower values produced a stricter, smaller reference region, where the surviving K562 reference shrank toward the densest centre of the (Annexin V, 7-AAD) cloud. This would narrow the viability quadrant, therefore causing more co-culture events to be classified as non-viable, which would systematically inflate the apparent cytotoxicity. Higher values produced a broader reference region and reduced that bias at the cost of admitting events from the apoptotic tails, resulting lower cytotoxicity. The optimum was identified at 95 % percent, balancing agreement between the manual consensus and within-replicate precision.

3. Optimized workflow parameters

The selected specifications from the optimized workflow, used as default parameters within the workflow are described in the following table (Table 2).

Table 2 Optimized workflow parameter specifications.

Workflow step	Parameter	Value	description
Initial scatter/ debris gate	FSC-A	2,4*10^6 to 1,0*10^7	Gate based on reference bead size for minimum cutoff
Initial scatter/ debris gate	SSC-A	0 to 1.0x10^7	
Morphology/singlet gate	gate2_method	ratio	Robust log-ratio gate method applied to log10(SSC-A / SSC-H)
Morphology/singlet gate	gate2_mad_mult	3.0	Events were retained within median ± 3x mean absolute deviation
Effector/target GFP separation	bw_adjust	0.05	Kernel density estimate (KDE) smoothing bandwidth multiplier
Effector/target GFP separation	min_distance	30	Minimum index distance between KDE peaks during peak/minimum detection
Reference proximity gate	method	Euclidean	kNN-density-based reference selection. The densest point in Annexin V / 7-AAD space is identified, then events closest by Euclidean distance are retained
Reference proximity gate	percent	0.95	Fraction of effector-only and target-only reference events retained after proximity selection
Reference proximity gate	kNN	3	Number of nearest neighbours used for kNN-density estimation
Quadrant gate derivation	gate_derivation	percentile	Annexin V and 7-AAD quadrant cutoffs, derived from reference population quantiles
Quadrant gate derivation	percentile_q	0.90	90th percentile used for percentile-based quadrant thresholding

Literature

Biau, Gérard, and Luc Devroye. 2015. *Springer Series in the Data Sciences Lectures on the Nearest Neighbor Method*. http://www.springer.com/series/13852.

Gerke, Oke. 2020. "Reporting Standards for a Bland-Altman Agreement Analysis: A Review of Methodological Reviews." *Diagnostics* 10(5).

Hahne, Florian, Nolwenn LeMeur, Ryan R. Brinkman, Byron Ellis, Perry Haaland, Deepayan Sarkar, Josef Spidlen, Errol Strain, and Robert Gentleman. 2009. "FlowCore: A Bioconductor Package for High Throughput Flow Cytometry." *BMC Bioinformatics* 10. doi:10.1186/1471-2105-10-106.

Leys, Christophe, Christophe Ley, Olivier Klein, Philippe Bernard, and Laurent Licata. 2013. "Detecting Outliers: Do Not Use Standard Deviation around the Mean, Use Absolute Deviation around the

Median." *Journal of Experimental Social Psychology* 49(4):764–66. doi:10.1016/j.jesp.2013.03.013.

Pihur, Vasyl, Susmita Datta, and Somnath Datta. 2009. "RankAggreg, an R Package for Weighted Rank Aggregation." *BMC Bioinformatics* 10. doi:10.1186/1471-2105-10-62.

Schober, Patrick, and Lothar A. Schwarte. 2018. "Correlation Coefficients: Appropriate Use and Interpretation." *Anesthesia and Analgesia* 126(5):1763–68. doi:10.1213/ANE.0000000000002864.

Watson, P. F., and A. Petrie. 2010. "Method Agreement Analysis: A Review of Correct Methodology." *Theriogenology* 73(9):1167–79.
